# Description of Male, Redescription of Female, Host Record, and Phylogenetic Position of *Haemaphysalis danieli*

**DOI:** 10.3390/pathogens11121495

**Published:** 2022-12-08

**Authors:** Iftikhar Ahmad, Shafi Ullah, Abdulaziz Alouffi, Mashal M. Almutairi, Mehran Khan, Muhammad Numan, Sher Zaman Safi, Lidia Chitimia-Dobler, Tetsuya Tanaka, Abid Ali

**Affiliations:** 1 Department of Zoology, Abdul Wali Khan University Mardan, Mardan 23200, Pakistan; 2 King Abdulaziz City for Science and Technology, Riyadh 12354, Saudi Arabia; 3 Department of Pharmacology and Toxicology, College of Pharmacy, King Saud University, Riyadh 11451, Saudi Arabia; 4 Faculty of Medicine, Bioscience and Nursing, MAHSA University, Jenjarom 42610, Malaysia; 5 Bundeswehr Institute of Microbiology, Munich 80937, Germany; 6 Laboratory of Infectious Diseases, Joint Faculty of Veterinary Medicine, Kagoshima University, Kagoshima 890-0065, Japan

**Keywords:** *Haemaphysalis danieli*, host, 16S rDNA, *cox1*, phylogeny

## Abstract

*Haemaphysalis* ticks are globally distributed with the greatest diversity in the Oriental region. This study aimed to primarily provide information on the morphology, host record, and preliminary phylogenetic position of a poorly known tick *Haemaphysalis danieli*. Herds comprised of goats and sheep were examined for this tick species in Upper Dir, Khyber Pakhtunkhwa, Pakistan. A total of 127 ticks, including males (n = 15, 11.8%) and females (n = 112, 88.2%), were collected, and morphologically identified as *H. danieli*. The morphological identification was confirmed through the 16S rDNA and *cytochrome c oxidase* (*cox1*) sequences. Phylogenetic analysis inferred based on 16S rDNA and *cox1* showed a close evolutionary relationship of *H. danieli* with a conspecific from China and an undetermined *Haemaphysalis* sp. from China and Anatolia. A total of 32/223 (14.3%) goats in two different herds were the only host infested by *H. danieli*. The earliest study provided the morphological description of *H. danieli* male, host record, and phylogenetic position. The information provided herein could assist in minimizing the knowledge gap regarding the systematic and taxonomy of *Haemaphysalis* species.

## 1. Introduction

*Haemaphysalis* species are found in highly variable climatic conditions in different zoogeographical regions, varying from humid, mild, cold winter, well-vegetated habitats to dry, hot summers [1,2]. The species of this genus are distributed throughout the world, with the most significant number recorded in the Oriental region. Some known species of the genus *Haemaphysalis* are also reported from the Palearctic and Ethiopian regions [1,2]. 

*Haemaphysalis danieli* Černý and Hoogstraal, 1977, belongs to the primitive subgenus *Allophysalis* of the Oriental and Palearctic region, adaptive to a high elevation of approximately 2000–4000 m (m) [2,3,4]. The species belonging to the subgenus *Allophysalis*, which are reported in the Asian mountain ranges and comprise *Haemaphysalis tibetensis* Hoogstraal, 1965 [5], *Haemaphysalis pospelovashtromae* Hoogstraal, 1966 [6], *Haemaphysalis warburtoni* Nuttall, 1912 [6], *Haemaphysalis garhwalensis* Dhanda and Bhat, 1968 [7], *H. danieli* [3], *Haemaphysalis kopetdaghica* Kerbabaev, 1962 [8], and *Haemaphysalis demidovae* Emelyanova, 1978 [4]. Teng (1980) has reported *Haemaphysalis xinjiangensis* as a valid species, while Kolonin (2009) considered this species as a synonym of *H. pospelovashtromae* [2]. Later, due to the lack of valid morphological and taxonomic evidence, it was not accepted as a valid species [2].

The immature (nymphal and larval) stages and a female tick of *H. danieli* were collected in 1970 from different northern regions of Pakistan: Swat (currently Chitral), Gilgit Baltistan (Phandar, Babusar, and Rama Valley), Hazara district (Gitidas and Khagan Valley), and Afghanistan: Wakhan area of Badakhshan province [3]. Different wild animals of the order Rodentia such as *Phodopus sungorus* Pallas, 1973, *Apodemus gurkha* Thomas, 1924, *Apodemus flavicollis* Melchior, 1834, *Hyperacrius wynnei* Blanford, 1881, *Marmota himalayana* Hodgson, 1841, *Cricetulus migratorius* Pallas, 1773, *Alticola roylei* Gray, 1842, *Marmota caudata* Geoffroy, 1844, and *Ochotona roylei* Ogilby, 1839, were suggested to be the hosts for the immature stages of *H. danieli*, while domestic and/or wild caprine and ovine were considered the probable hosts for the adults of this tick [3]. Most of the *Haemaphysalis* species are associated with wild animals, but changes in their feeding behavior have been noticed. These species switched from wild hosts to domestic animals like cattle, sheep, and goats [4,9,10].

Morpho-molecular and phylogenetic analyses are among the best methods adopted for understanding the identification and systematics of known and rare and/or poorly known tick species [4,11]. To our knowledge, there is no comprehensive study on the morphology of male, host records, and phylogenetic analyses of *H. danieli* ticks. Therefore, the current study aimed to describe the *H. danieli* male ticks, host record, and preliminary phylogenetic analysis.

## 2. Materials and Methods

### 2.1. Ethical Statement

Ethical approval was obtained from the Advanced Studies and Research Board (Dir/A&R/AWKUM/2022/9396) of the Faculty of Chemical and Life Sciences, Abdul Wali Khan University Mardan, KP, Pakistan. Oral permissions were taken from the owners of animals or herds.

### 2.2. Study Area

**Figure 1:**
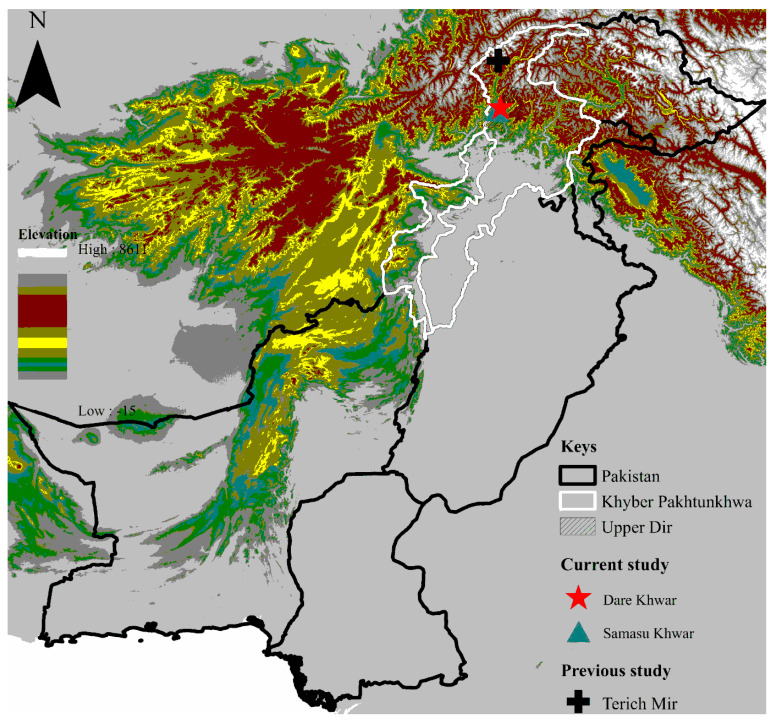
Elevation-based map showing the collection sites where tick specimens were collected.

The province of Khyber Pakhtunkhwa (KP) shares its border through the international boundary with Afghanistan in the northwest and the north, and both countries are connected by the Hindu Kush range. This study was conducted in the district Upper Dir (35°35′34.7″ N 71°57′40.2″ E), comprised of two collection regions: Dare Khwar (35°20′49.0″ N 71°49′40.1″ E) and Samasu Khwar (35°20′25.1″ N 71°48′38.0″ E), KP, Pakistan. The physiography of the area includes the terrain of rugged, gently steep slopes. District Upper Dir is part of the subtropical dry and to some moist temperate regions, representing four seasons: long, cold, and severe winter, and short summer, spring, and autumn. The study district is located in a hilly, mountainous, and highly elevated area (~3000 m); average temperature (−7 °C to 35 °C), high relative humidity above 70%, and precipitation (770 mm). The Global Positioning System (GPS) was used to find the geo-coordinates of the collection points. The elevation-based map was designed through ArcGIS 10.3.1 (ESRI, Redlands, CA, USA) (Figure 1).

### 2.3. Tick Collection and Preservation

Small ruminants, including 223 goats and 168 sheep kept individually and/or transhumant/nomadic herds in the tick collection sites, were examined for ticks. Ticks were directly collected from the skin by fine-tipped tweezers during the summer in July 2022. Tick specimens were stored in 1.5 mL tubes and shifted to the Department of Zoology, Abdul Wali Khan University, Mardan. The collected tick specimens were rinsed with distilled water, followed by 70% ethanol to remove any tissue debris, and preserved in 100% ethanol in well-labeled plastic tubes for further molecular analyses.

### 2.4. Morphological Identification of Ticks

Tick specimens were morphologically identified and confirmed up to the species level using a stereomicroscope (StereoBlue-euromex SB.1302-1, Arnhem, The Netherlands) with 3.5–135×, using a standard taxonomic identification key based on different morphological characters [3]. Tick specimens were photographed with 50–200× magnification using a Keyence microscope (Illinois, VHX 900F, Itasca, IL, USA).

### 2.5. DNA Extraction and PCR

A total of 15 tick specimens (two males and thirteen females) were subjected to genomic DNA extraction for molecular identification. The tick specimens were crushed with sterilized pestles in 1.5 mL Eppendorf tubes. The genomic DNA was extracted from each tick specimen using a phenol-chloroform protocol [12] per the standard guidelines. The DNA pellet was hydrated by adding 30 µL of PCR water “nuclease-free”. The extracted genomic DNA was quantified using NanoDrop (Nano-Q, Optizen, Daejeon, Korea).

Extracted genomic DNA was amplified through a conventional PCR (GE-96G, BIOER, Hangzhou, China) targeting mitochondrial 16S rRNA and *cox1* partial fragments (Table 1). Each PCR reaction mixture was prepared in a 25 μL contained: 12 μL of Dream*Taq* MasterMix (Thermo Fisher Scientific, Inc., Waltham, MA, USA), 1 μL of each primer (10 μM), 3 μL (100 ng/μL) template DNA, and 8 μL PCR water. Thermocycler conditions and primers are shown in Table 1. Each PCR reaction run contained *Haemaphysalis bispinosa* Neumann, 1897 DNA and PCR water as a positive and negative control, respectively. The PCR amplified DNA was run on a 2% agarose gel stained with ethidium bromide (Thermo Fisher Scientific, Inc., Waltham, MA, USA) and visualized on a Gel Documentation (BioDoc-It™ Imaging Systems UVP, LLC, Upland, CA, USA).

### 2.6. DNA Sequencing and Phylogenetic Analyses

The PCR amplified amplicons were purified using the GeneClean II Kit (Qbiogene, Il-lkirch, France) following the manufacturer’s protocol. A total of 30 amplified or purified products (one for 16S rDNA and one for *cox1* from each tick specimen) were sequenced bidirectionally in a commercial company (Macrogen, Inc., Seoul, Republic of Korea) by the Sanger sequencing method. The obtained sequences were trimmed and assembled in SeqMan V. 5.0 (DNASTAR, Inc., Madison, WI, USA) to remove low-quality nucleotide sequences and primer regions. The purified sequences were subjected to BLAST (Basic Local Alignment Search Tool: https://blast.ncbi.nlm.nih.gov/Blast.cgi, accessed on 4 September 2022) [15] using the searching tool NCBI (National Center for Biotechnology Information). The homologous sequences were downloaded from NCBI in FASTA format for phylogenetic analysis. These sequences were aligned in BioEdit Sequence Alignment Editor V. 7.0.5 (Raleigh, NC, USA) [16] using ClustalW Multiple alignments [17] with the obtained and an outgroup sequence (*Haemaphysalis punctata*). The phylogenetic trees for 16S rDNA and *cox1* sequences were constructed according to the Maximum Likelihood method in MEGA-X (Molecular Evolutionary Genetics Analysis) with a 1000 bootstrapping value and Kimura 2-parameter model [18]. The coding sequences (*cox1*) were aligned by MUSCLE [19]. The obtained sequences formed the dataset’s final positions.

## 3. Results

### 3.1. Collected Ticks and Host Record

**Figure 2:**
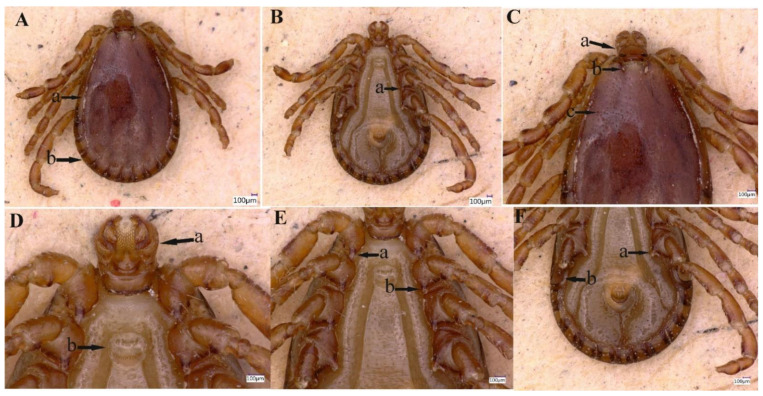
Male *Haemaphysalis danieli*. (**A**) Lateral groove (a), festoon (b). (**B**) Coxa III, spur (a). (**C**) Capitulum dorsally (a), cervical groove (b), punctuations (c). (**D**) Capitulum ventrally (a), genital aperture (b). (**E**) Spur of coxae I (a) and II (b). (**F**) Coxa IV, spur (a) and spiracular plate (b).

**Figure 3:**
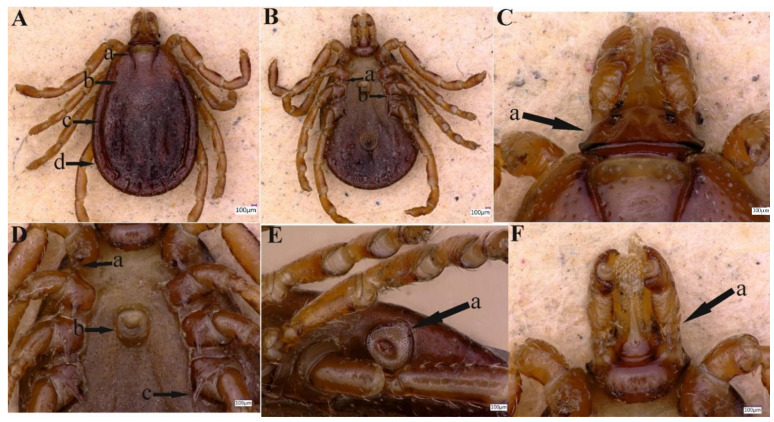
Female *Haemaphysalis danieli*. (**A**) Cervical groove (a), scutum (b), lateral groove (c) and festoon (d). (**B**) Spur of coxae II (a) and III (b). (**C**) Capitulum dorsally (a). (**D**) Coxa I, spur (a), genital aperture (b) and coxa IV, spur (c). (**E**) Spiracular plate (stigma) (a). (**F**) Capitulum ventrally (a).

A total of 127 ticks were collected and all were morphologically identified as *H. danieli* (Figure 2 and Figure 3), comprised of males (n = 15, 11.8%), females (n = 112, 88.2%), and no immature stages of the tick were collected. All *H. danieli* ticks were found only on goats in two different herds containing goats and sheep. These two herds were found at two different locations (Dare Khwar and Samasu Khwar) of Upper Dir, separated by a distance of approximately 4–5 km (km). Nearly four ticks/host, a total of 32/223 (14.3%) goats, were infested by *H. danieli* ticks.

### 3.2. Description of Haemaphysalis danieli

#### 3.2.1. Description of Male

Idiosoma: Ornamentation is indistinct on the conscutum, with reddish brown to dark reddish color; body is oval shape with length (excluding capitulum) 2.1 mm, greatest width 1.6 mm, (Figure 2A (a,b). Conscutum is pear-shaped, about twice as long as wide, and is widest at the level of the fourth leg, Figure 2A (a,b); punctuations are shallow, mostly small, and of medium size, distributed slightly dense and not very uniform, Figure 2C (c). Scapulae are short and blunt; the margins are narrow and shallow; cervical grooves are deep, linear anteriorly and converging posteriorly; lateral grooves are narrow and long, enclosing the first two anterior festoons; eyes absent; 11 festoons ranging from 0.13 to 0.17 mm in width and 0.17 to 0.2 mm in length, Figure 2A (a,b). Stigmas are oval-elongated with a narrower part dorsally and rounded macula located on the antero-inferior side, Figure 2F (b); anus with “Y” anal groove, “Y” tail reaching the festoon and the lateral arms exceed the anus anteriorly (Figure 2B). The genital apron is located between the coxa II, Figure 2D (b).

Gnathosoma: Length from apices to posterior margin of the basis 0.37 mm; the basis capituli is 1.8 times wider than long (including the cornua), with the posterior margin being straight and having thick, short, and blunt cornua, Figure 2C (a). Palpi are elongated and clavate, with a length of four articles as follows: article 1, 0.07 mm; article 2, 0.17 mm. The distal end of article 2 is noticeably wider, being 1.5 times longer than the third article. Article 3 was 0.12 mm, without external spur; article 4 is in the apical pit, visible ventrally; hypostome is slightly shorter than the palpi, having length 0.24 mm and width at base 0.12 mm; dental formula is 5/5, distally a rosette is visible, Figure 2D (a).

Legs were short and robust (Figure 2A,B). All coxae have a well-developed inner spur, similar in size and shape, Figure 2E (a,b), slightly longer than the width of its base, narrow and blunt or firmly pointed at the end, and slightly curved to the outside, Figure 2E (a,b) and Figure 2F (a). Tarsus I tapers distally, measuring a length of 0.29 mm, with a clear, oval area on the dorsum of tarsi I, Haller’s organ.

Chaetotaxy: Small and tiny hairs can be observed on the palps and legs.

#### 3.2.2. Redescription of Female

Idiosoma: Scutum lacks ornamentation, having a dark brown color. This tick possesses an elliptical-shaped body with a maximum length (excluding capitulum) of 2.86 mm and a maximum width of 2.09 mm (Figure 3A (a–d). Scutum is pear-shaped, slightly longer than width, is widest between the second and third legs, narrows backward, with a blunt end, Figure 3 (b); punctuations are thick and deep, distributed densely, but not uniform, Figure 3 (b). Scapulae are round; cervical grooves are deep, linear anteriorly, and converging posteriorly, reaching about 1/3 of the scutum; eyes are absent; 11 festoons with average width 0.22–0.24 mm and length 0.20–0.60 mm, Figure 3A (a,b). Stigma is pear-shaped, tapering towards the outside, Figure 3E (a); anus with “Y” shaped anal groove, “Y” tail extending towards the central festoon and the lateral arms diverging anteriorly and exceeding the anus, (Figure 3B). The genital aperture is located between the coxae II and III with a U-shaped, Figure 3D (b).

Gnathosoma: Length from the anterior top to the posterior bottom 0.66 mm (Figure 3C); basis capituli is about 3 times wider than long*;* cornua absent, Figure 3C (a); the porose areas are moderate size, oval, and distantly spaced, with a shallow depression between them, Figure 3C (a). Ventrally, basis capituli is wide and short, with the posterior edge arced, Figure 3F (a). Palpi elongated and clavate, with a length of four articles as follows: article 1, 0.13 mm, article 2, 0.29 mm, narrowing from front to back, the distal end of article 2 is wider, 1.8–1.9 times longer as the third article 3, 0.16 mm, the posterior margin is straight, article 4, 0.1 mm (located in the third article pit) and only visible ventrally, Figure 3C (a). Hypostome is slightly shorter than the palpi with a length of 0.54 mm and width at base 0.25 mm, dental formula is 5/5, end with a crone, Figure 3F (a). For more detailed information on female *H. danieli*, see the standard key proposed by the expert [3].

Legs: The main features of each leg are the same as those of male ticks, Figure 3A–F.

Chaetotaxy: Fine hairs can be seen on the palps and legs.

### 3.3. Molecular Analyses

DNA was extracted from all 15 *H. danieli* ticks. The nucleotide sequences of the expected size, one 16S rDNA and one *cox1* per tick, were obtained. The sequences belonging to the same partial fragments were found to be identical. Two consensus sequences were obtained, including 16S rDNA (390 bp) and *cox1* (675 bp), which were considered for further analyses. By BLAST analysis, the 16S rDNA showed 98.48–99.24% identity with *H. danieli* (NC062065 and MH394440) from China, followed by 98.70–99.72% with *Haemaphysalis* sp. (MZ463296 and MG021192) from Turkey and China, and 90.95% with *H. tibetensis* (OM049539, OM368296, and NC062066) from China. The BLAST analysis of *cox1* displayed 97.92% identity with *H. danieli* (NC062065), followed by 89.75% with *H. tibetensis* (OM368296, ON783071, OM049539, and NC062066) reported from China.

The consensus DNA sequences of *H. danieli* were submitted to GenBank under accession numbers OP435750 (16S rDNA) and OP435801 (*cox1*).

### 3.4. Phylogenetic Analysis

**Figure 4:**
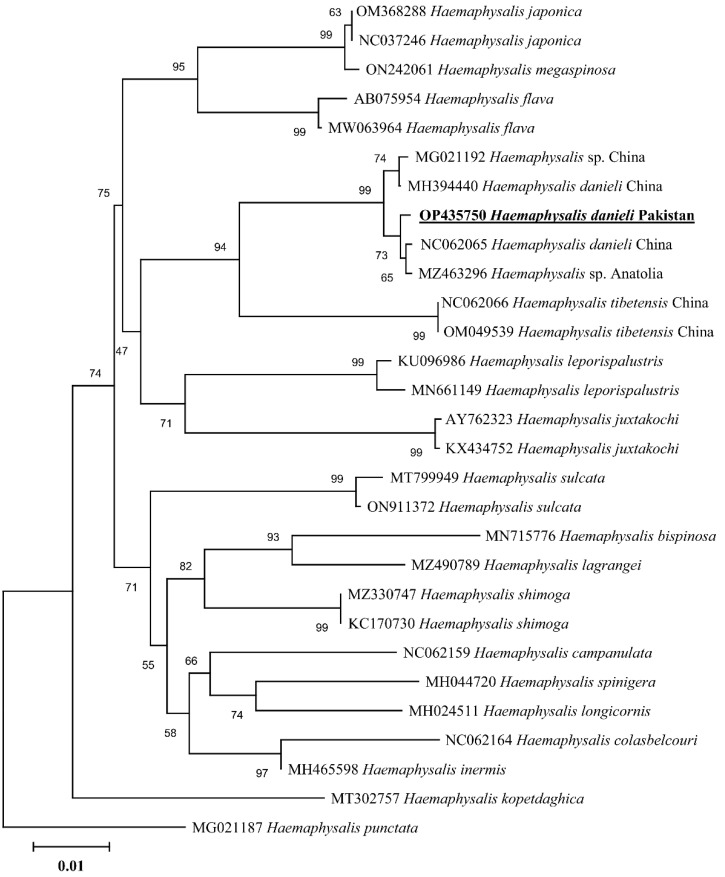
Maximum-likelihood phylogenetic analysis of *Haemaphysalis danieli* based on 16S rDNA sequence. The sequences are represented by their GenBank accession numbers followed by the names of species and countries (when applicable). The branch lengths show the number of substitutions per site inferred based on the scale displayed. The obtained sequence in the present study is indicated in bold and underlined.

**Figure 5:**
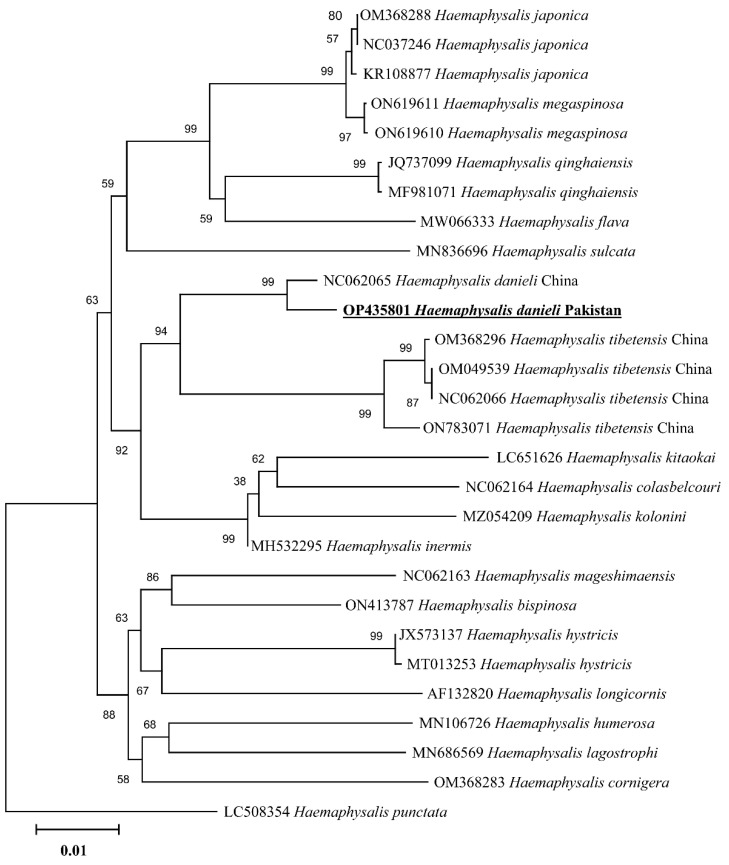
Maximum-likelihood phylogenetic analysis of *Haemaphysalis danieli* based on *cox1* sequence. The sequences are represented by their GenBank accession numbers followed by the names of species and countries (when applicable). The branch lengths show the number of substitutions per site inferred based on the scale displayed. The sequence obtained in the present study is indicated in bold and underlined.

In the phylogenetic analysis based on 16S rDNA (Figure 4), the *H. danieli* of the present study clustered with the same species and an undetermined *Haemaphysalis* sp. compared to the phylogenetic tree based on *cox1* (Figure 5), where *H. danieli* was clustered only with the same species. In both phylogenetic analyses, *H. danieli* appeared in a monophyletic branch with different species (*H. tibetensis*).

## 4. Discussion

Genus *Haemaphysalis* counts the second most diversified genus of existing tick fauna, containing 176 species [2,20,21]. With the existence of large diversity in ticks of the Oriental region and the lack of systematic knowledge associated with terms of precise identification and categorization, these ticks have mostly remained unidentified accurately. Herein, we described the male morphology, host record, and preliminary phylogenetic position of *H. danieli*.

*Haemaphysalis danieli* collected from goats represents the first confirmed host and male tick record in the country, while formerly, only a female tick was collected from vegetation inhabited by wild ibex and many rodents [3]. Herein, female ticks outnumbered male ticks, which could be attributed to unequal hatching and mortality rates [22,23,24], and such tick species may have a selective advantage [25]. Furthermore, it could also depend on the season and area of collection. Comparing the collection points of current and previous studies suggests that it might be due to habitat expansion, or the migration of hosts associated with this tick [3], as currently, this tick has been collected from goats in Upper Dir about 102 km away from the previous collection done in the Tirich Mir valley. In our current study, all ticks were collected at an altitude reaching up to ∼3000 m in the western Himalayas in view of the fact that *H. danieli* prefers rocky biotopes at high altitudes [3], as all members of the subgenus *Allophysalis* were reported from high terrains (1600–4000 m) of Himalayan and Hindu Kush ranges [4]. Some life stages of species belonging to the subgenus *Allophysalis* are unknown because of inhabiting such harsh topography, and thus being difficult for researchers to find [2].

Descriptions of male and female *H. danieli* revealed that moderate-sized coxal spurs exhibit hook-like or spiny shapes to firmly attach to the host [4]. As a member of the subgenus *Allophysalis*, *H. danieli* females possess elongated palpi, pointing towards the primitive group of ticks [26]. The description of *H. xinjiangensis* developed confusion as a new species in China [27], confirmed by Hoogstraal as a new species of the subgenus *Allophysalis* [4]. Kolonin (2009) believed that *H. xinjiangensis* is a synonym of *H. pospelovashtromae*, but to support his view, he did not provide any conclusive evidence. Therefore, Guglielmone et al. [2,28] treated *H. xinjiangensis* as a synonym of *H. danieli*.

When morphology becomes unreliable, the rapid and precise identification of any life stage of a tick can be achieved through molecular approaches by using phylogenetic analysis [29,30,31,32,33,34], targeting mitochondrial genes like 16S rRNA and *cox1* [33,34,35,36]. In the phylogenetic trees, the obtained partial sequences of 16S rDNA and *cox1* clustered with the species from the same subgenus, e.g., *H. danieli* and *H. tibetensis* reported from China. Furthermore, *H. tibetensis* clustered as a sister clade to *H. danieli* in the phylogenetic trees, which authenticated the relation between the two species and classified them into the same subgenus *Allophysalis* [4]. Additionally, the morphological description has been validated by the current phylogenetic analyses.

## 5. Conclusions

This study rediscovered a poorly known *H. danieli* tick, found the host record, and genetically characterized it for the first time. The study confirmed that *H. danieli* is endemic to the mountains of the Hindu Kush-Himalayan (HKH) region located at the junction of the Oriental and Palearctic regions. Besides contributing to the genomic data of *Haemaphysalis* ticks enlisted in the Oriental region, this study may assist in understanding the systematic and taxonomy of *Haemaphysalis* species.

## Figures and Tables

**Table 1:** Primers and PCR conditions used for the amplification of ticks.

Target Genes	Sequences (5′-3′)	Amplicon Size	PCR Conditions	References
16S rRNA	16S+1-CCGGTCTGAACTCAGATCAAGT 16S−1-GCTCAATGATTTTTTAAATTGCTG	460 bp	95 °C 3 min, 40 × (95 °C 30 s, 55 °C 60 s, 72 °C 1 min), 72 °C 7 min	[13]
*cox1*	HC02198-TAAACTTCAGGGTGACCAAAAAATCA LCO1490-GGTCAACAAATCATAAAGATATTGG	710 bp	95 °C 30 s, 40 × (95 °C 30 s, 48 °C 30 s, 72 °C 1 min), 72 °C 5 min	[14]

## Data Availability

Details regarding data supporting the reported results can be found https://www.ncbi.nlm.nih.gov/nuccore/?term= (accessed on 4 September 2022).
